# Supplementary material for: Ramon Flour (Brosimum alicastrum Swartz) Ameliorates Hepatic Lipid Accumulation, Induction of AMPK Phosphorylation, and Expression of the Hepatic Antioxidant System in a High-Fat-Diet-Induced Obesity Mouse Model
Source: Antioxidants (Basel). 2023 Nov 2;12(11):1957. doi: 10.3390/antiox12111957 (PMC10669741; doi:10.3390/antiox12111957)
Supplement: Supplementary file 1 [file antioxidants-12-01957-s001.zip › antioxidants-2665838-supplementary.pdf]

Table S1. Description of composition of experimental diets (g/100g diet).

Nutrient	Source	AIN-93	AIN-93 adjusted 25% Ramon flour	HFD	HFD adjusted 25% Ramon flour
**Carbohydrate**		**62.9**	**62.9**	**41.6**	**41.6**
	Cornstarch	39.7	28.8	26.3	41.5
	Maltodextrin	13.3	9.39	8.73	14.8
	Sucrose	10	7.11	6.61	3.72
	Ramon flour	-	18.2	-	18.2
**Protein**		**20**	**20**	**24**	**24**
	Casein	20	17.1	24	21.1
	Ramon flour	-	2.9	-	2.9
**Lipid**		**7**	**24**	**7**	**24**
	Soy oil	7	6.7	7	6.7
	Lard	-	-	17	17
	Ramon flour	-	0.3	-	0.3
**Fiber**		**5**	**5.3**	**5**	**5.3**
	Cellulose	5	-	5	-
	Ramon flour	-	5.3	-	5.3
**Mineral**	Mineral mix	3.5	3.5	3.5	3.5
**Vitamin**	Vitamin mix	1	1	1	1
	L-Cystine	0.3	0.3	0.3	0.3
	Choline bitartrate	0.25	0.25	0.25	0.25
	TBHQ	0.0014	0.0014	0.0014	0.0014

Table S2. Sequencies used for mRNA expression by SYBER GREEN assays.

**Gen**	**Forward**	**Reverse**
Acox1	GCCATTCGATACAGTGCTGTGAG	CCGAGAAAGTGGAAGGCATAGG
Cpt1	GCACTGCAGCTCGCACATTACAA	CTCAGACAGTACCTCCTTCAGGAAA
Fas	ATGCACACTCTGCGATGAAG	CAGTGTTCACAGCCAGGAGA
Sod2	TAACGCGCAGATCATGCAGCTG	AGGCTGAAGAGCGACCTGAGTT
Cyclophilin	CAGGGGAGATGGCACAGGAG	CGGCTGTCTGTCTTGGTGCTCTCC
